# Cardiometabolic Risk in Psoriatic Arthritis: A Hidden Burden of Inflammation and Metabolic Dysregulation

**DOI:** 10.3390/metabo15030206

**Published:** 2025-03-18

**Authors:** Mislav Radić, Andrej Belančić, Hana Đogaš, Marijana Vučković, Yusuf Ziya Sener, Seher Sener, Almir Fajkić, Josipa Radić

**Affiliations:** 1 Department of Internal Medicine, Division of Rheumatology, Allergology and Clinical Immunology, Center of Excellence for Systemic Sclerosis in Croatia, University Hospital of Split, 21000 Split, Croatia; mislavradic@gmail.com; 2 Internal Medicine Department, School of Medicine, University of Split, 21000 Split, Croatia; 3 Department of Basic and Clinical Pharmacology with Toxicology, Faculty of Medicine, University of Rijeka, Braće Branchetta 20, 51000 Rijeka, Croatia; 4 Department of Neurology, University Hospital of Split, 21000 Split, Croatia; hana.dogas@gmail.com; 5 Department of Internal Medicine, Division of Nephrology and Dialysis, University Hospital of Split, 21000 Split, Croatia; mavuckovic@kbsplit.hr; 6 Department of Pediatric Rheumatology, Sophia Children’s Hospital, Erasmus University Medical Center, 3000 CB Rotterdam, The Netherlands; yzsener@yahoo.com.tr; 7 Department of Cardiology, Thoraxcenter, Erasmus University Medical Center, 3000 CB Rotterdam, The Netherlands; kzl_seher@hotmail.com; 8 Department of Pathophysiology, Faculty of Medicine, University of Sarajevo, 71000 Sarajevo, Bosnia and Herzegovina; almir.fajkic@mf.unsa.ba

**Keywords:** psoriatic arthritis, cardiometabolic risk, adipokines, systemic inflammation, oxidative stress, insulin resistance, dyslipidemia

## Abstract

Psoriatic arthritis (PsA) is a chronic inflammatory disease that extends beyond musculoskeletal and dermatologic involvement to elevate cardiometabolic risk. Emerging evidence highlights the critical role of systemic inflammation in metabolic dysregulation, accelerating insulin resistance, dyslipidemia, and oxidative stress, all of which contribute to the increased burden of cardiovascular disease in PsA. This review explores the intricate interplay between inflammatory mediators—such as tumor necrosis factor-alpha (TNF-α), interleukin-6 (IL-6), and interleukin-17 (IL-17),—adipokine imbalances, and lipid metabolism abnormalities, all of which foster endothelial dysfunction and atherosclerosis. The dysregulation of adipokines, including leptin, adiponectin, and resistin, further perpetuates inflammatory cascades, exacerbating cardiovascular risk. Additionally, the metabolic alterations seen in PsA, particularly insulin resistance and lipid dysfunction, not only contribute to cardiovascular comorbidities but also impact disease severity and therapeutic response. Understanding these mechanistic links is imperative for refining risk stratification strategies and tailoring interventions. By integrating targeted immunomodulatory therapies with metabolic and cardiovascular risk management, a more comprehensive approach to PsA treatment can be achieved. Future research must focus on elucidating shared inflammatory and metabolic pathways, enabling the development of innovative therapeutic strategies to mitigate both systemic inflammation and cardiometabolic complications in PsA.

## 1. Introduction

Psoriatic arthritis (PsA) is a chronic, immune-mediated inflammatory disease primarily affecting the musculoskeletal system and skin, but its impact extends far beyond joint and dermatologic involvement [1]. Characterized by synovitis, enthesitis, dactylitis, and axial disease, PsA imposes a significant burden on physical function and quality of life [2]. Traditionally classified within the spectrum of spondyloarthropathies, PsA is now increasingly recognized as a systemic disorder with widespread metabolic and cardiovascular implications [3,4]. Recent evidence highlights that the inflammatory milieu in PsA is a key driver of metabolic dysregulation, influencing the pathogenesis of insulin resistance, dyslipidemia, and endothelial dysfunction, ultimately accelerating cardiovascular disease (CVD) risk.

Cardiometabolic comorbidities in PsA have emerged as critical contributors to patient morbidity and mortality. Patients with PsA exhibit a substantially increased risk of cardiovascular events, including myocardial infarction and stroke, compared to the general population [5,6,7]. Systemic inflammation, mediated by cytokines, such as tumor necrosis factor-alpha (TNF-α), interleukin-6 (IL-6), and interleukin-17 (IL-17), plays a pivotal role in modulating lipid metabolism, insulin signaling, and oxidative stress, thereby fostering an environment conducive to accelerated atherogenesis. Furthermore, adipokine imbalances, including elevated leptin and resistin levels alongside decreased adiponectin, perpetuate pro-inflammatory pathways and contribute to metabolic disturbances in PsA patients [8,9].

Despite the well-established association between systemic inflammation and cardiometabolic risk, these comorbidities remain underrecognized and undertreated in routine PsA management. Traditional cardiovascular risk assessment models often underestimate the burden in PsA due to their failure to account for chronic inflammation as an independent risk factor [3,4,8,9]. Given these gaps, there is an urgent need for a paradigm shift in PsA management, integrating targeted immunomodulatory therapies with comprehensive metabolic and cardiovascular risk stratification.

**Figure 1:**
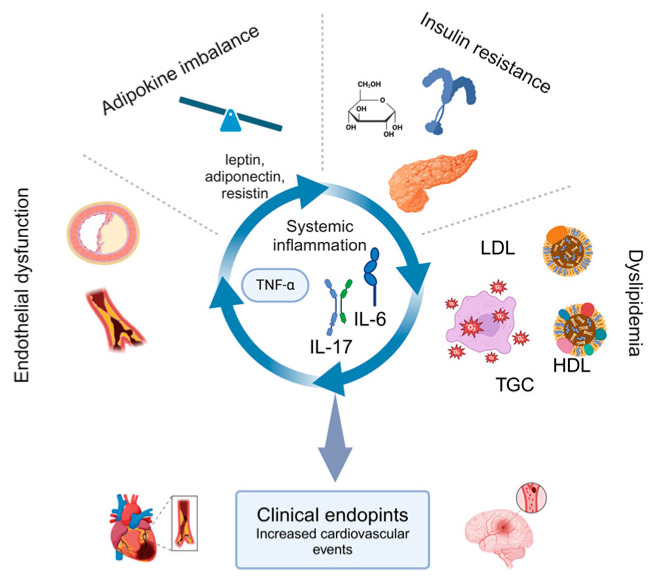
Interplay between inflammation, metabolic dysregulation, and cardiovascular risk in psoriatic arthritis.

This review aims to elucidate the mechanistic links between systemic inflammation in PsA and its metabolic and cardiovascular sequelae. By exploring the interplay between pro-inflammatory cytokines, adipokine dysregulation, oxidative stress, and endothelial dysfunction, we provide a comprehensive overview of how chronic inflammation drives metabolic and vascular pathology in PsA (Figure 1). Furthermore, we highlight the implications for clinical practice, emphasizing the need for early screening, personalized risk assessment, and innovative therapeutic strategies to mitigate both systemic inflammation and cardiometabolic complications in PsA patients.

## 2. Systemic Inflammation and Its Cardiometabolic Implications

PsA is characterized by the presence of chronic inflammation and immune dysregulation [10,11]. Beyond its musculoskeletal and dermatological manifestations, PsA has significant cardiometabolic implications, including an increased risk of CVD, metabolic syndrome, and type 2 diabetes mellitus (T2DM). The systemic inflammatory milieu in PsA plays a pivotal role in driving these comorbidities, necessitating a deeper understanding of the underlying mechanisms and therapeutic approaches [7].

The development of atherosclerosis is a multifaceted process influenced by chronic inflammation through several mechanisms [12]. Inflammation leads to metabolic dysregulation, which exacerbates the risk of CVD and metabolic disorders. This dysregulation is predominantly driven by the persistent activity of pro-inflammatory cytokines, such as TNF-α, IL-6, and IL-17 [13]. These cytokines lead to a cascade of metabolic dysfunctions, such as insulin resistance, adipokine imbalance, and vascular inflammation [13].

Patients with PsA are at higher risk of CVD, metabolic syndrome, and other comorbidities, including T2DM, hypertension, and obesity [7]. Studies have indicated that PsA patients exhibit a two-to threefold elevated risk of developing CVD compared to the general population. This elevated risk is primarily attributable to persistent systemic inflammation, which is associated with accelerated atherosclerosis, endothelial dysfunction, and insulin resistance. A study by González-Gay et al. [14] demonstrated that the risk of myocardial infarction in PsA patients was higher than in patients with rheumatoid arthritis and significantly higher than in age- and sex-matched controls without PsA. Furthermore, PsA has been associated with an augmented prevalence of coronary artery disease.

### 2.1. Pro-Inflammatory Cytokines and Insulin Resistance

TNF-α and IL-6 have been demonstrated to play crucial roles in the impairment of insulin signaling [15]. TNF-α promotes serine phosphorylation of insulin receptor substrate-1 (IRS-1), thereby impeding its capacity to transduce insulin signaling [16]. This, in turn, results in a reduction in glucose uptake by muscle and adipose tissues, thereby contributing to the development of hyperglycemia and insulin resistance. IL-6 further exacerbates this dysfunction by altering mitochondrial functions and increasing the accumulation of fatty acids in mitochondria, impairing insulin sensitivity [17,18].

In PsA, adipose tissue displays increased infiltration of immune cells, including macrophages and T-cells, which collectively amplifies the inflammatory response [19]. The elevated secretion of TNF-α and other cytokines from these cells perpetuates insulin resistance, linking PsA to increased susceptibility to type 2 diabetes mellitus [20]. The chronic inflammatory environment also suppresses the activity of insulin receptors in hepatic and skeletal muscle tissues, further aggravating systemic hyperglycemia and insulin resistance.

### 2.2. Adipokine Imbalance

Adipokines, bioactive molecules secreted by adipose tissue, play a critical role in metabolic regulation (Table 1) [21]. PsA-associated inflammation disrupts the adipokine balance by decreasing levels of anti-inflammatory adiponectin and increasing levels of pro-inflammatory leptin [22]. Adiponectin, a key player in the adipokine network, has been shown to improve insulin sensitivity and exert protective effects against vascular inflammation [23]. However, its suppression in PsA contributes to the development of insulin resistance and endothelial dysfunction [22]. Conversely, elevated leptin levels stimulate the production of pro-inflammatory cytokines, such as TNF-α, perpetuating the cycle of inflammation and metabolic dysregulation [24].

Visceral adiposity, a hallmark of PsA patients with metabolic syndrome, exacerbates this imbalance [25]. The excess adipose tissue becomes a reservoir for pro-inflammatory cytokines, thereby enhancing the systemic inflammatory burden [25]. This condition establishes a feed-forward loop, where inflammation worsens adipokine imbalance and vice versa, creating a metabolic state predisposed to insulin resistance and cardiovascular events.

### 2.3. Vascular Inflammation

Chronic systemic inflammation in PsA promotes vascular inflammation, a critical factor in the development of atherosclerosis [12]. Pro-inflammatory cytokines, such as TNF-α and IL-6, induce endothelial dysfunction by reducing nitric oxide (NO) bioavailability, impairing vasodilation, and increasing oxidative stress [26]. Furthermore, these cytokines stimulate the production of adhesion molecules, thereby facilitating the recruitment of immune cells to the vascular wall. As a result, endothelial damage and macrophage infiltration accelerate plaque formation, and increase the risk of cardiovascular events such as myocardial infarction and stroke [12,26].

IL-17 also plays a significant role in vascular inflammation [27]. It has been observed to promote smooth muscle proliferation and enhance the deposition of low-density lipoproteins (LDL) within the vascular intima. This contributes to the formation of vulnerable plaques that are more likely to rupture and carry a higher risk of acute cardiovascular events. The synergistic action of IL-17, TNF-α, and IL-6 amplifies the inflammatory response, exacerbating vascular injury and promoting atherogenesis [28].

### 2.4. Inflammation and Lipid Metabolism

Systemic inflammation in PsA has also been demonstrated to disrupt lipid metabolism, thereby contributing to the development of dyslipidemia [29]. Elevated levels of pro-inflammatory cytokines have been demonstrated to interfere with normal lipid transport and utilization. For instance, TNF-α downregulates lipoprotein lipase activity, leading to elevated triglyceride levels [30]. Conversely, IL-6 has been observed to promote the production of acute-phase proteins, such as C-reactive protein (CRP) and serum amyloid A, which replace apolipoproteins on high-density lipoprotein (HDL). This transformation impairs the reverse cholesterol transport function of HDL, reducing its cardioprotective properties [31].

Inflammation may lower LDL cholesterol levels, and dyslipidemia is more prominent in patients with active disease. Changes in the composition of lipids, such as a reduction in HDL3 subfraction and an increase in dense LDL subfraction, can lead to atherogenic dyslipidemia, even if LDL cholesterol levels are lower [8]. Patients with PsA frequently exhibit an atherogenic lipid profile, characterized by elevated triglycerides, reduced HDL cholesterol, and increased small, dense LDL particles [32]. Consequently, alterations in lipid levels in conjunction with vascular inflammation significantly elevate the risk of atherosclerosis and cardiovascular events in patients with PsA.

### 2.5. Hyperuricemia

High uric acid levels are common in patients with PsA and can be a risk factor for CVD. Obesity is also common in patients with PsA and contributes to high uric acid levels [33]. In addition, increased production of uric acid due to the overgrowth and breakdown of keratinocytes plays a role in high uric acid levels in PsA [34]. Uric acid triggers inflammatory pathways in liver cells and increases oxidative stress in adipocytes, leading to adipokine imbalance [35]. It is also well documented that hyperuricemia is related to hypertension and atherosclerotic CVD [36].

## 3. Role of Adipokines in PsA and Cardiovascular Risk

PsA is a chronic inflammatory condition also associated with a significantly elevated risk of CVD, with patients demonstrating a 43% higher likelihood of cardiovascular events, such as myocardial infarction and stroke, compared to the general population [37]. This increased risk is not solely attributable to traditional cardiovascular risk factors; rather, chronic systemic inflammation plays a pivotal role in exacerbating vascular dysfunction and promoting atherosclerosis [37]. Central to this inflammatory process are adipokines, bioactive molecules secreted by adipose tissue, which exhibit both pro-inflammatory and anti-inflammatory properties, thereby influencing PsA pathogenesis and cardiometabolic risk [38].

Adipokines, such as leptin, adiponectin, and resistin, play dual roles in PsA and cardiovascular health. Leptin, primarily known for regulating energy balance, exhibits pro-inflammatory effects by promoting the production of cytokines, like TNF-α and IL-6, both of which are implicated in PsA pathogenesis and atherogenesis [39]. Elevated leptin levels have been observed in PsA patients, correlating with disease activity and systemic inflammation, thereby contributing to endothelial dysfunction and increased cardiovascular risk [38,39]. Furthermore, leptin has been shown to induce endothelial cell proliferation and angiogenesis, processes that exacerbate vascular pathology in PsA [39].

Conversely, adiponectin, an adipokine with anti-inflammatory and cardioprotective properties, is typically reduced in PsA patients. Lower adiponectin levels are associated with increased disease activity and subclinical myocardial dysfunction, as evidenced by decreased global longitudinal strain (GLS) in echocardiographic assessments [37]. Adiponectin enhances insulin sensitivity and exerts anti-atherogenic effects by inhibiting vascular smooth muscle cell proliferation and reducing endothelial adhesion molecule expression [38,39]. The “adiponectin paradox”, wherein elevated levels are observed in advanced heart failure, complicates its role, suggesting that low adiponectin levels may be an early marker of cardiometabolic risk in PsA [37].

Resistin, another pro-inflammatory adipokine, is elevated in PsA and psoriasis patients and is linked to insulin resistance and metabolic syndrome components, such as hyperglycemia and dyslipidemia [40]. The resistin concentration has been found to correlate with disease severity in psoriasis and PsA, further implicating its role in chronic inflammation and atherogenesis [39]. While resistin levels correlate with traditional cardiovascular risk factors, their direct association with carotid intima-media thickness, a marker of subclinical atherosclerosis, remains unclear [40]. Nevertheless, the presence of elevated resistin suggests a role in perpetuating low-grade systemic inflammation and vascular dysfunction in PsA.

Obesity, prevalent among PsA patients, further complicates the interplay between adipokines and cardiovascular risk. Obese PsA patients exhibit higher disease severity, increased joint involvement, and elevated inflammatory markers, such as CRP [41]. The pro-inflammatory milieu created by excess adipose tissue, rich in adipokines, like leptin and resistin, exacerbates systemic inflammation and contributes to endothelial dysfunction and atherosclerosis [39,42]. Interestingly, while obesity is a known risk factor for PsA development, it also predicts poorer treatment responses, particularly to TNF inhibitors, underscoring the importance of weight management in mitigating both disease activity and cardiovascular risk [41].

The interrelationship between adipokines and cardiovascular risk in PsA extends to their influence on myocardial function. Subclinical myocardial dysfunction, detected via advanced echocardiographic techniques, like speckle tracking echocardiography (STE), is prevalent in PsA patients even in the absence of overt cardiovascular disease [37]. Elevated levels of IL-17A, a pro-inflammatory cytokine associated with PsA disease activity, correlate with impaired myocardial strain, suggesting a link between systemic inflammation, adipokine dysregulation, and cardiac dysfunction [37]. Moreover, decreased adiponectin levels are independently associated with reduced GLS, highlighting the cardioprotective role of this adipokine in preserving myocardial function [37].

Therapeutic interventions targeting inflammatory pathways in PsA also modulate adipokine levels and may influence cardiovascular outcomes. For instance, treatment with apremilast, a phosphodiesterase 4 inhibitor, has been shown to reduce inflammatory cytokines and adipokines, improve endothelial function, and decrease insulin resistance in PsA patients with metabolic syndrome [43]. These findings suggest that controlling systemic inflammation not only ameliorates PsA symptoms but also mitigates cardiovascular risk by modulating adipokine profiles and improving vascular health.

In conclusion, adipokines play crucial roles in the pathogenesis of PsA and its associated cardiovascular risk. Their dual functions in promoting or mitigating inflammation underscore the complex interplay between metabolic and immune pathways in PsA. Adipokines interact with immune cells, contributing to the inflammatory network and influencing vascular function, immune regulation, and glucose metabolism, thereby serving as key players in the pathogenesis of PsA and its cardiometabolic complications [39]. Addressing adipokine dysregulation through targeted therapies and lifestyle interventions, such as weight management, holds promise in reducing both disease burden and cardiovascular morbidity in PsA patients. Future research should continue to elucidate the precise mechanisms by which adipokines influence cardiovascular risk and explore their potential as biomarkers for disease activity and therapeutic response in PsA.

## 4. Insulin Resistance and Metabolic Dysregulation

PsA, classically associated with joint and skin involvement, is garnering increasing attention for its intimate association with other metabolic diseases, such as T2DM and metabolic syndrome (MetS). Peripheral tissues show a reduced sensitivity to insulin, leading to lower glucose uptake from the blood into cells, representing a common aspect of T2DM and MetS. The relationship between PsA and insulin resistance is complex and interlinked, stemming from common pathophysiology, such as systemic inflammation and metabolic derangement, and underscoring a significant overlap in pathogenic pathways [22].

Various epidemiological studies confirm the data that PsA patients are at a higher risk of developing T2DM and MetS, which highlights the considerable metabolic burden that they experience. Chronic inflammation is one of the hallmarks of PsA and plays a role in the development of dyslipidemia, visceral obesity, and prothrombotic states, all of which increase the risk of metabolic disorders and their complications. This coexistence of PsA, along with altered metabolism, generates a challenging scenario for therapeutic interventions and cardiovascular risk factor reduction and contributes towards the significantly increased risk of and their associated morbidity and mortality [44].

Many studies further proved that compared to the general population, T2DM is more prevalent among patients with PsA. A cohort study by Eder et al. reported a standardized prevalence ratio (SPR) of 1.43 for T2DM in PsA patients compared to the general population in Ontario (Canada), with a significant increase in prevalence in the PsA group [45]. Similarly, Queiro et al. reported among PsA patients for which 13.8% had T2DM compared to 5% in a control group without inflammatory conditions, with an odds ratio (OR) of 2.8, indicating a markedly increased prevalence of T2DM among subjects with PsA [46]. Furthermore, Dubreuil et al. reported an incidence rate of diabetes among PsA patients of 7.3 per 1000 person-years. After correcting the influences of body mass index (BMI), smoking, and alcohol intake, the study yielded an adjusted hazard ratio (HR) of 1.33, so the minority population appears to be at higher risk for T2DM overall [47].

Some populations with an increased burden of T2DM and PsA share inflammatory pathways, genetic predisposition, and shared risk factors (e.g., obesity). A greater incidence of T2DM has been previously described in individuals with PsA versus the general population, with obesity being an important contributor to this association [48]. Genetic studies have also revealed overlapping loci between psoriasis and T2DM, which indicate a genetic susceptibility that also could be implicated in PsA. Pathways, such as nuclear factor kappa-light-chain-enhancer of activated B cells (NF-κB) signaling, exemplify the potentially shared genetic architecture, which is involved in immune-mediated and metabolic diseases [49]. Also, demographic variables, such as late-onset psoriasis (after the age of 40) and hypertension, as well as other comorbidities, increase T2DM risk. The high prevalence of T2DM has also been reported in some ethnic groups and has been reported to be high in American Indian, African, Hispanic, and Asian populations [46].

Several shared pathophysiological mechanisms of insulin resistance in relation to PsA are primarily mediated via chronic inflammation and metabolic dysregulation. Chronic inflammation in PsA, characterized by enhanced levels of cytokines and adipokines, impairs the insulin signaling pathway, thus leading to insulin resistance [22]. Central to the underlying pathology are key pro-inflammatory factors, such as TNFα, IL-6, and interleukin-1β (IL-1β). IL-1β, for instance, has shown a link in promoting insulin resistance by inhibiting the action of insulin on various cellular functions from targeting keratinocytes, which is particularly relevant in psoriasis and PsA [50]. This inflammatory background is mediated by systemic metabolic alteration, thereby strengthening the link of PsA with insulin resistance.

Further supporting the association between chronic inflammation and glucose derangements are studies exploring the significant overlap between inflammatory pathways in psoriasis and PsA. Data demonstrate that psoriasis is associated with increased insulin resistance and systemic inflammation, which may contribute significantly to this association [51]. Among those implicated cytokines, IL-17 has been emphasized as an important mediator potentially bridging psoriasis with hyperglycemia; thus, similar mechanisms might exacerbate glucose imbalance in PsA [52]. Potentially, plasma glucose increases in chronic inflammation are due to increased hepatic glucose production as in other inflammatory conditions [53]. It suggests that the chronic inflammatory state in PsA may also negatively affect glucose homeostasis, which could explain the metabolic derangements commonly observed in these patients.

Adipokines, including leptin, adiponectin, resistin, and others, regulate inflammation and metabolic processes. In PsA, dysregulated levels of these adipokines have been reported, potentially stimulating systemic inflammation and insulin resistance. High levels of leptin found in obesity are known to be pro-inflammatory and have been demonstrated to correlate with disease activity and inflammation in patients with PsA. Likewise, resistin has been linked to heightened disease severity and inflammation, particularly in PsA patients on anti-TNF-α therapy [54,55]. Conversely, another cytokine, adiponectin, is lower in the serum of PsA patients than in healthy individuals and may act to increase inflammation by causing metabolic dysfunction as well [20]. Furthermore, patients with PsA have also been reported to have MetS more frequently than patients with only psoriasis, and these differences are associated with increased levels of pro-inflammatory adipokines and greater insulin resistance, as assessed by higher HOMA-IR levels [54]. These findings highlight the roles of adipokines in both exacerbating inflammatory processes and contributing to the metabolic dysregulation seen in PsA, emphasizing their importance as potential therapeutic targets.

Endothelial dysfunction is common in PsA and positively relates to disease activity and insulin resistance, indicating the strong link between inflammation and metabolic dysregulation [56]. C3 complement level is a major predictor of whole-body insulin sensitivity in PsA patients, suggesting that immune system components are directly involved in metabolic processes [57]. Additionally, molecular studies showed overlapping signaling pathways involving Rap1, PI3K-Akt, and cGMP-PKG, which are implicated in the pathogenesis of psoriasis and insulin resistance in T2DM, that offer better insights into the mechanistic connections between PsA, inflammation, and metabolic dysregulation [58].

Hypertension and dyslipidemia, common in PsA patients, are well-established risk factors that contribute to MetS and increase the risk of T2DM in combination with obesity [46]. Furthermore, psoriasis severity is correlated with an increased risk of insulin resistance. Patients with severe psoriasis are substantially at a higher risk of developing T2DM than their counterparts with less severe disease, thus indicating a dose-response association between the area of their skin involvement and metabolic risk [59].

Insulin resistance, highly correlated with chronic inflammation in PsA, has been demonstrated to impair the effectiveness of biological therapies. Patients with high levels of insulin resistance have lower response rates to biologic therapies, as measured by improvements in Psoriasis Area and Severity Index (PASI) and Physician Global Assessment (PGA) scores [60]. This attenuated effect might be due to insulin resistance’s metabolic and inflammatory dysregulation characteristics. In addition, this condition is associated with an increased cardiovascular risk and other metabolic comorbidities, which adds to the complexity of the overall management of PsA. Chronic inflammation in PsA, caused by insulin resistance, causes endothelial dysfunction and exacerbates atherosclerosis, thus worsening cardiovascular morbidity and mortality [56]. These findings further highlight the importance of developing a multitargeted treatment approach that focuses on the inflammatory manifestations of PsA and related metabolic comorbidities to optimize the overall benefit to the patient [44].

Untreated insulin resistance in PsA patients can result in significant complications, mainly due to its connection with metabolic syndrome and chronic inflammation. If left untreated, the most concerning outcome of insulin resistance is the development of T2DM, associated with a complex chain of late complications, such as microangiopathy (retinopathy, nephropathy, and neuropathy) and macroangiopathy (increased cardiovascular events). Chronic inflammation in PsA also exacerbates these risks, contributing to atherosclerosis and cardiovascular disease [61,62]. Moreover, dyslipidemia, noted for elevated triglycerides and LDL cholesterol. associated with insulin resistance and subsequent hyperinsulinemia, is markedly raised and enhances cardiac risk [48]. In PsA patients, the comorbidities of metabolic syndrome and sustained inflammation are the interplay between different factors that increase the risk of developing cardiovascular ailments, including ischemic heart disease, cerebrovascular disease, and peripheral vascular disease among those with insulin resistance [62]. Additionally, chronic inflammatory conditions associated with PsA can enhance other metabolic disorders, namely obesity and hypertension, which are both contributors to metabolic syndrome and have been shown to worsen insulin resistance [61]. Such inflammatory and metabolic dysregulation induces a vicious cycle, contributing to the mutual advancement of PsA and its comorbidities. To improve outcomes, this cycle must be broken through comprehensive management, including inflammation and metabolic health.

## 5. Dyslipidemia and Altered Lipid Metabolism

PsA is associated with an increased prevalence of cardiovascular morbidity and cardiovascular risk factors compared to the general population [63]. The results of a recent meta-analysis of 39 studies and a combined sample of more than 150,000 participants show that hyperlipidemia is the third most common comorbidity in PsA participants, after diabetes and arterial hypertension [64], and that hyperlipidemia is more common in PsA participants than in the general population [63]. Together with arterial stiffness, as evidenced by a high intima-media thickness of the carotid artery [65].

Compared to psoriasis alone and the healthy control group, LDL levels were highest in the PsA group but did not reach statistical significance. On the other hand, statistical significance was achieved for oxidized LDL (oxLDL) in this group (*p* < 0.001). In addition, patients with PsA had the highest total cholesterol/HDL cholesterol and LDL cholesterol/HDL cholesterol ratios (respectively, *p* < 0.05), as shown in a study of 93 participants with psoriasis and PsA and 60 healthy controls [32].

Oxidative stress caused by systemic inflammation and metabolic disorders exacerbates lipid abnormalities by promoting lipid peroxidation [66]. Adherent monocytes differentiate into macrophages in the subendothelial layer, which take up oxLDL via scavenger receptors and convert them into lipid-laden foam cells [67]. This process can also occur in vascular smooth muscle cells (VSMCs) and lead to VSMC-derived foam cells. oxLDL can stimulate migration, proliferation, calcification, the switch from contractile to synthetic cells, and apoptosis [66]. In macrophages, oxLDL triggers inflammation, activation of inflammasomes, and polarization of macrophages and promotes platelet aggregation [68,69]. Persistent inflammation can lead to plaque erosion, atherothrombosis, tissue ischemia and acute cardiovascular events [69].

Consequently, dysregulated lipids contribute to oxidative stress and enhance lipid peroxidation, thereby increasing vascular damage in this damaging cycle. For this reason, lipid oxidation and chronic inflammation interact to increase the risk of cardiovascular morbidity and mortality in patients with PsA.

Myeloid differentiation factor 2 (MD2) has been shown to be responsible for TLR4 activation and inflammation by directly binding to ox-LDL, which triggers the formation of the MD2/TLR4 complex and the proinflammatory TLR4-MyD88-NFκB cascade, opening a perspective for potential new therapeutic options for the treatment of atherosclerosis [70].

In addition, the results of the drug target study, which used the Mendelian randomization strategy and data from population-based genome association studies, showed that genetic inhibition of proprotein convertase subtilisin/kexin type 9 (PCSK9) correlated with a reduced risk of PsA (odds ratio [OR]: 0.51; 95% CI 0.14–0.88; *p* < 0.01), which raises the possibility of extending the use of PCSK9 inhibitors beyond their known lipid-lowering use [71].

In summary, there is a close interplay between dyslipidemia and altered lipid metabolism with chronic inflammation and oxidative stress in PsA, which promotes lipid peroxidation, foam cell formation, and cardiovascular risk. However, further insights are needed to identify the precise molecular mechanisms behind the interplay of dyslipidemia, inflammation, and oxidative stress in this population and to test other newer therapeutic strategies, such as MD2/TLR4 pathway inhibitors and PCSK9 inhibitors, as well as other potential molecules.

## 6. Oxidative Stress and Endothelial Dysfunction

PsA is also characterized by the combination of psoriasis and arthritis, as well as inflammatory cytokines and oxidative stress, which are key factors in the pathogenesis of PsA and its comorbidities. PsA patients have an increased prevalence of cardiovascular comorbidities, with higher rates of peripheral vascular disease, congestive heart failure, atherosclerosis, ischemic heart disease, cerebrovascular disease, and hypertension. The reported prevalence ratios for control groups for these conditions are 1.3 and 1.6 [72]. In addition, PsA is associated with increased intimal medial thickness, a marker of subclinical atherosclerosis. This parameter has been found to correlate with disease activity and conventional cardiovascular risk factors, including hypertension and hyperlipidemia [72].

Oxidative stress is a key pathogenic factor of atherosclerosis, and a condition characterized by oxidative stress, such as PsA, represents a high-risk situation for cardiovascular events. The pathophysiological mechanism includes an overproduction of reactive oxygen species (ROS) through multiple enzymatic pathways, including NADPH oxidase, xanthine oxidase, and uncoupled endothelial nitric oxide synthase (eNOS) [73,74,75]. Biomarkers of oxidative stress, such as malondialdehyde (MDA) and advanced oxidation protein products (AOPP), are elevated in PsA patients, while antioxidant enzyme activities, such as superoxide dismutase (SOD) and glutathione peroxidase (GPx), are reduced, correlating with disease severity [76]. The oxidative stress in PsA is also linked to mitochondrial dysfunction and hypoxia in the synovial tissue, which further increases ROS production and contributes to joint damage [77].

Endothelial cells are sensitive to the detrimental effects of oxidative stress, which compromises their functional characteristics and induces activation. This endothelial dysfunction is mediated by decreased NO bioavailability, an important molecule for promoting vascular health and preventing atherosclerosis [78]. Thus, oxidative modifications of lipoproteins, primarily LDL, generate oxLDL, which plays a vital role in atherogenesis. Crucially, oxLDL plays a significant role in driving endothelial activation, monocyte adhesion and infiltration, and smooth muscle cell proliferation, all of which are critical events leading to the development of atherosclerotic plaques [79]. In addition, the activity of paraoxonase 1 (PON1), an antioxidative enzyme associated with high-density lipoprotein, is reduced in psoriatic disease [76]. The diminished PON1 activity has been associated with increased disease activity and cardiovascular burden, particularly in the case of PsA, indicating that oxidative stress and inflammation might be more closely related to cardiovascular risk factors in patients with PsA [80]. The increase in oxidized lipids and systemic inflammation further increases cardiovascular risk with increased CRP levels in affected individuals [81].

The relationship between oxidative stress and chronic inflammation in PsA leads to a self-perpetuating cycle that further compounds injuries caused by the endothelium and drives the evolution of atherosclerosis. In psoriasis and psoriatic arthritis, the chronic inflammatory environment raises pro-inflammatory cytokines and chemokines, exacerbating the production of ROS and aggravating endothelial dysfunction [82,83]. Cytokines and oxidative stress are intimately related in that cytokines stimulate the generation of ROS, and oxidative stress modulates cytokines. For instance, releasing oxidized proteins, such as peroxiredoxin-2, can serve as an inflammatory signal, exacerbating the inflammatory response [84].

Some key inflammatory cytokines have been found to underlie the oxidative damage implicated in the etiology of PsA through multiple interconnected mechanisms. Pro-inflammatory cytokines, IL-6, IL-17, IL-23, and TNF-α, are significantly elevated, contributing to the inflammatory milieu associated with PsA. These cytokines activate immune cells, including neutrophils and macrophages, increasing their ability to produce ROS in oxidative bursts [85,86,87].

IL-6 is a pro-inflammatory cytokine involved in several inflammatory and autoimmune conditions, including PsA. While the referenced studies do not directly establish the role of IL-6 in mediating oxidative damage in PsA itself, they shed light on the involvement of oxidative stress and inflammation in IL-6 activity in related processes. Upregulation of the angiotensin II type 1 receptor by IL-6 has been demonstrated to facilitate oxidative stress in vascular tissues and stimulate ROS production [88]. The oxidative stress reduces NO bioavailability, a critical factor in maintaining endothelial function, thereby contributing to vascular dysfunction [89].

This mechanism highlights the ability of IL-6 bioactivity to induce oxidative stress in tissues where it is overexpressed. In addition, IL-6 has been correlated with impaired redox homeostasis in skeletal muscle, enhancing free radical production and accumulation and resulting in a pro-oxidative milieu [90]. These findings suggest that IL-6 may mediate oxidative stress in several different tissues. Additionally, IL-6 trans-signaling via the soluble IL-6 receptor was also shown to play a pathological role in cardiovascular dysfunction associated with inflammatory arthritis. It is possible that a similar mechanism could play a role in cardiovascular-related complications in PsA [91].

In the specific context of PsA, there is not sufficient direct evidence that IL-6 is indeed performing oxidative damage in PsA but that an association exists between IL-6 expression and DNA damage in PsA patients, where it appears to be possible that IL-6 mediates oxidative stress [92]. However, the exact mechanisms of IL-6-mediated oxidative damage in PsA still need to be explored.

Oxidative damage and inflammation mediated by the IL-17 signaling pathway play essential roles in the pathogenesis of PsA. IL-17, as a pro-inflammatory cytokine, contributes to the recruitment and activation of immune cells, which secrete ROS, causing oxidative stress. A pronounced elevation in systemic IL-17 has been shown to induce systemic endothelial dysfunction and vascular oxidative stress, which have been linked with increased cardiovascular risk in psoriasis and PsA. IL-17 activates endothelial cells, releasing inflammatory cytokines and adhesion molecules, thus augmenting vascular inflammation [93].

IL-17 is involved in oxidative stress through different mechanisms and has been studied for this effect. IL-17A has been demonstrated to induce ROS production, contributing to endothelial dysfunction and vascular inflammation in psoriasis-like conditions [94]. These findings have implications for PsA, given overlapping inflammatory pathways between the two conditions. Moreover, IL-17A promotes the proliferation of pro-inflammatory Th17 cells, which can produce IL-17 without stimulating IL-23, thereby exacerbating inflammation in a pro-inflammatory milieu in PsA [95]. In addition, IL-17A has been linked to metabolic reprogramming that promotes oxidative stress. Specifically in psoriasis, IL-17A has been shown to promote metabolic reprogramming of keratinocytes towards increased glycolysis and lipid uptake, associated with increased ROS production [96]. These observations concerning psoriasis may, at least in part, reflect the phenomenon underpinning IL-17A-induced oxidative stress and probably include PsA because of the shared pathophysiological features of both conditions.

IL-23 is an important cytokine involved in the IL-23/IL-17 axis responsible for Th17 cell differentiation and maintenance. These T-helper cells release pro-inflammatory cytokines, including IL-17 and granulocyte-macrophage colony-stimulating factor (GM-CSF), that play a crucial role in the unique inflammatory processes associated with PsA [97]. The IL-23 pathway drives myeloid-derived cell subset expansion, such as neutrophils, which have been implicated in the cutaneous and articular manifestations of PsA. These immune cells produce ROS and cause oxidative stress and subsequent tissue damage. Moreover, IL-23 regulates bone metabolic processes by upregulating osteoclastogenesis and contributes to joint destruction by inducing bone resorption [98,99].

Interestingly, IL-23 has other functions besides its role in IL-17 signaling. Given the evidence of IL-23-independent IL-17 production, PsA may still involve IL-23 as a distinct, independent contributor to Th17 pathology [100]. This underscores the complex role of IL-23 in promoting oxidative damage by directly inducing immune cells and indirectly through cytokine signaling and bone remodeling processes.

IL-23 has been implicated in the modulation of oxidative stress, a hallmark of endothelial dysfunction. IL-23 potentiates these cardiovascular inflammatory and oxidative stress responses within the setting of myocardial I/R injury, which ultimately increases infarct size and cardiomyocyte apoptosis. This is evidenced by markedly increased levels of stress markers, including lactate dehydrogenase (LDH), creatine kinase, malondialdehyde levels, and decreased activity of SOD, which are indicative of oxidative stress [101]. This inflammatory response is further enhanced by increased IL-17A, TNF-α, IL-6, and other mediators involved in developing endothelial dysfunction. IL-23 promotes inflammation through the IL-23/IL-17 axis, which can cause endothelial dysfunction in atherosclerosis. IL-23 is upregulated in patients suffering from carotid atherosclerosis and is positively correlated with the production of IL-17, subsequent inflammation, and finally, the progression of atherosclerotic disease [98]. This inflammatory environment may induce oxidative stress and further compromise endothelium function.

Additionally, IL-23 has been implicated in modulating oxidative stress pathways and regulating cellular senescence in fibroblasts. It can regulate the expression of GADD45a and the p38/MAPK pathway, which means it can attenuate senescence caused by oxidative stress. This shows that its role in the oxidative stress response may not be as simple [102].

TNF-α is an essential mediator in PsA pathogenesis and a major contributor to oxidative damage through various pathways. TNF-α signaling promotes ROS generation, pivotal in propagating oxidative stress and inflammatory processes. One primary mechanism is activating NADPH oxidase, a key source of cellular ROS. TNF-α stimulates the phosphorylation of the NADPH oxidase subunit p47phox, which then interacts with TRAF4 to activate the enzyme complex. PI3K/AKT pathway activation leads to increased ROS production, activating downstream kinases, such as ERK1/2 and p38 MAPK, further promoting inflammatory responses [103]. An alternative pathway involves a cPLA2-dependent cascade, leading to TNF-α-induced production of ROS. Arachidonic acid metabolism via 5-lipoxygenase produces leukotriene B4 (LTB4), potentiating ROS formation. This cascade depends on the small GTPase Rac, an essential determinant for TNF-α-induced ROS production [104].

TNF-α inhibitors (etanercept) have been shown to improve endothelial function among patients with PsA and psoriasis, possibly through decreasing oxidative stress and inflammation. They enhance the antioxidant action of HDLs and promote endothelial-dependent vasodilation. Such therapeutic benefits emphasize that TNF-α plays an essential role in mediating the vascular and inflammatory changes in PsA [105].

TNF-α rapidly induces ROS production in primary human keratinocytes, leading to degradation of IκB and nuclear translocation of NF-κB p65. This generates ROS and mTOR pathway-mediated pro-inflammatory cytokines. ROS induces this process, but ROS are not directly required to activate NF-κB [106].

Additionally, TNF-α regulates oxidative tricarboxylic acid (TCA) cycle flux in endothelial cells to modulate mitochondrial metabolism. This is achieved via pyruvate dehydrogenase kinase 4 (PDK4) degradation by the Lon protease, leading to increased pyruvate dehydrogenase activity. The resulting accumulation of citrate and acetyl-CoA promotes histone acetylation, which supports the transcription of pro-inflammatory genes [107]. The complex pathways highlight the critical contribution of TNF-α to oxidative stress and inflammation in PsA. They furthermore elucidate possible therapeutic avenues for reducing oxidative damage and preventing disease progression in this pathological entity.

This relational complexity associates oxidative stress and inflammation with cardiovascular comorbidities in PsA, which highlights the requirement of diagnostic, therapeutic methods as approachable targeting means for these categories of interrelated processes. Targeting oxidative stress seems promising, but PsA is a multifactorial disease that would best respond to an integrative approach involving anti-inflammatory and antioxidant interventions. Further insights into the molecular pathways that mediate these interactions, and their mechanisms would enrich our knowledge and pave the way for newer therapeutic strategies to control disease severity and its sequelae. Insights like these could eventually pave the way for tailored therapeutics to enhance clinical outcomes in those suffering from PsA.

## 7. Clinical Implications: Screening and Risk Management

Individuals diagnosed with PsA exhibit an elevated risk of developing CVD when compared to the general population [108]. A multitude of studies have demonstrated that CVD and stroke manifest more frequently in individuals with PsA, even after adjusting for conventional cardiovascular risk factors [109]. Many cohort studies have consistently demonstrated that patients with PsA experience an accelerated process of atherosclerosis [110]. This increased risk appears to be associated not only with conventional risk factors, such as hypertension, dyslipidemia, and smoking, but also with the inflammatory nature of PsA itself. In view of the higher cardiovascular and metabolic risks concomitant with PsA, periodic screening for cardiometabolic risk factors is imperative for the timely identification of increased risk, thus facilitating the implementation of preventive measures.

### 7.1. Screening for Cardiovascular Risk

There are several tools, including the Framingham Risk Score, American College of Cardiology/American Heart Association (ACC/AHA) Risk Calculator, QRISK3, Reynolds Risk Score, Atherosclerotic Cardiovascular Disease (ACD) Risk Calculator, and Systemic Coronary Risk Estimation (SCORE2), that can be utilized to estimate the cardiovascular risk in general population [111,112]. However, there are no unique tools for cardiovascular risk assessment in PsA and other inflammatory diseases. Recent ESC guidelines suggest the use of updated SCORE2 in patients with inflammatory disease, acknowledging that these patients have higher cardiovascular risk than estimated [112]. Moreover, some residual cardiovascular risk factors, such as lipoprotein (a), are increased in inflammatory conditions, and they are not included in existing cardiovascular risk estimating tools. Therefore, current cardiovascular risk scores underestimate the cardiovascular risk in patients with inflammatory diseases [113]. A comprehensive screening for cardiovascular risk factors, the majority of which are also included in cardiovascular risk assessment tools, should be performed in patients with PsA to implement preventive measures for modifiable risk [114]. The regular measurement of cholesterol and triglyceride levels can facilitate the determination of cardiovascular risk. Electrocardiography (ECG) and other cardiac tests to monitor heart health can be useful, especially in individuals at risk [114].

### 7.2. Risk Management

#### 7.2.1. Lifestyle Changes

The adoption of a healthy lifestyle, encompassing regular physical activity, a well-regulated diet, smoking cessation, and effective weight management, is critical in mitigating cardiovascular risk in PsA patients [115].

Lifestyle changes are the mainstay of cardiovascular risk reduction in patients with inflammatory diseases. Smoking cessation is potentially the most effective preventive measure, and its benefits are substantial at all ages [112]. There is conflicting data regarding the increased rate of smoking in patients with PsA, but smoking is a well-defined risk factor for psoriasis [116]. Therefore, smoking cessation should be recommended in all patients.

Guideline-based dietary recommendations should be given to patients with concomitant hypertension and diabetes. Glucocorticoids cause sodium, water retention, and impair glucose metabolism. Therefore, salt restriction and a diabetic diet should be advised in patients treated with glucocorticoids [117]. Anti-inflammatory diets, characterized by a diet abundant in omega-3 fatty acids, have been shown to be beneficial in both the management of arthritis and the reduction of cardiovascular disease risk [118]. Applying the Mediterranean diet may be reasonable as it reduces the risk of cardiovascular events in the general population, although this has not been confirmed in patients with PsA [116].

Obesity is associated with disease flares and worse outcomes in PsA. It is also a hallmark risk factor for CVD [116]. Obesity also impairs the treatment response in PsA. Weight loss strategies, including dietary modification and bariatric surgery, are beneficial in both PsA-related outcomes and cardiovascular risk reduction [41]. Glucagon-like peptide-1 receptor agonists (GLP1-RA) have been shown to be associated with improved cardiovascular outcomes, weight loss, and reduced inflammation and may be considered as first-line therapy in patients with PsA and diabetes or obesity [119,120]. Moreover, the introduction of GLP1-RA may provide additional benefits for other comorbidities, such as metabolic dysfunction-associated steatotic liver disease (MASLD), which is also more prevalent in PsA patients [121,122].

Regular exercise has been shown to improve joint function and cardiovascular health [123]. Physical activity is reduced in patients with PsA because of joint-related complaints, and exercise may worsen joint pain [116]. The benefits of physical activity in reducing disease activity and cardiovascular risk outweigh the small increased risk of enthesitis [123]. Both functional and resistance exercise improve PsA activity, and there is no specific recommendation for the type of exercise [124]. Aerobic exercise has been shown to reduce the risk of CVD, suggesting that it could be the suitable form of exercise for patients with PsA [125].

#### 7.2.2. Management of Traditional Cardiovascular Risk Factors

Hypertension is present in 20–25% of patients with PsA, and this high prevalence rate is possibly due to higher rates of obesity and uric acid levels. Blood pressure measurement is important in all patients with PsA as many of the cases are treated with non-steroidal anti-inflammatory drugs (NSAIDs) or glucocorticoids that may cause increased blood pressure [126,127]. Blood pressure target and threshold for treatment is the same as in the general population. Renin angiotensin system inhibitors and calcium channel blockers should be preferred primarily as diuretics lead to derangement in metabolic parameters and beta-blockers may trigger psoriatic flares [127]. Restriction of salt intake is recommended and a level of lower than 1500 mg sodium per day seems reasonable, especially in patients receiving glucocorticoids [117].

The increased rate of dyslipidemia in PsA is associated with future cardiovascular events. In patients diagnosed with PsA who are at high cardiovascular risk, the administration of statin therapy may prove effective in reducing the incidence of cardiovascular events [128]. Beyond their lipid-lowering effects, statins also possess anti-inflammatory properties and reduce the risk of cardiovascular events in PsA patients, particularly those with elevated CRP levels [129]. LDL cholesterol target should be determined based on cardiovascular risk as assessed with recent risk scores. It is assumed that the existing cardiovascular risk estimation tools underestimate cardiovascular risk in patients with PsA. Although the estimated cardiovascular risk is recommended to be multiplied by 1.5 in patients with rheumatoid arthritis, there is not any unique advice for PsA [112]. The administration of low-dose aspirin may also be considered as a means of preventing cardiovascular events in patients with high cardiovascular risk [128].

Fasting glucose or hemoglobin A1c (HbA1C) is recommended for the screening of patients with PsA for the presence of insulin resistance or diabetes [130]. Glucose lowering agents with proven cardiovascular benefits and weight-loss effects, such as sodium-glucose cotransporter 2 inhibitors (SGLT2i) and GLP1-RA, should be preferred primarily in diabetic PsA cases [131]. Glucocorticoids impair glucose metabolism, nevertheless, alternative anti-inflammatory treatments, such as anti-TNF-α and apremilast, have been shown to possess the potential to enhance glucose metabolism [132]. Consequently, restriction of calorie and sugar intake is recommended during corticosteroid treatment [117].

#### 7.2.3. Management of Inflammation

Given that inflammation itself contributes to cardiovascular and metabolic risk, it is imperative to assess and manage PsA disease activity. The Disease Activity Score for Psoriatic Arthritis (DAPSA) and Psoriatic Arthritis Disease Activity Score (PASDAS) are tools that can be used to assess the degree of inflammation and disease severity [133]. It is imperative to note that active disease, particularly in patients exhibiting elevated levels of inflammation, has been associated with an augmented risk of cardiovascular disease.

The treatment of PsA has historically centered on the management of joint inflammation and the enhancement of physical function [134]. However, in recent years, there has been an increasing recognition of the need to also address cardiometabolic risks due to the increased cardiovascular risk in PsA patients. Achieving effective management of systemic inflammation is imperative to reduce cardiovascular morbidity and prevent the development of atherosclerotic complications [135]. The anti-inflammatory treatment includes NSAIDs, glucocorticoids, and conventional or biologic DMARDs [136,137].

### 7.3. Medical Treatment

#### 7.3.1. Non-Steroidal Anti-Inflammatory Drugs

NSAIDs are the first-line treatment for PsA cases with mono/oligoarthritis, enthesitis, or axial disease [138]. NSAIDs, particularly strong cyclooxygenase 2 inhibitors, are associated with increased blood pressure, heart failure exacerbation, and thromboembolic events. They may also impair kidney functions. Therefore, blood pressure and kidney functions should be closely monitored in long term treatment with NSAIDs [139].

#### 7.3.2. Glucocorticoids

Glucocorticoids are frequently prescribed in the treatment of PsA; however, they are also associated with hypertension, obesity, insulin resistance, and dyslipidemia, which increase cardiovascular and metabolic risk [140,141]. It is therefore incumbent upon clinicians to closely monitor for the emergence of these adverse effects and to endeavor to minimize glucocorticoids use through the administration of alternative medications, such as conventional or biologic disease-modifying antirheumatic drugs (DMARDs), when feasible. Patients should pursue the abovementioned dietary recommendations to mitigate insulin resistance and hypertension.

#### 7.3.3. Conventional Synthetic Disease-Modifying Antirheumatic Drugs

Conventional synthetic DMARDs have been utilized as the primary treatment for PsA for an extended period, with the objective of managing disease activity and inflammation [65]. The utilization of conventional synthetic DMARDs, including methotrexate, sulfasalazine, and leflunomide, has been demonstrated to contribute to the reduction of joint damage and the enhancement of overall outcomes [142]. However, the cardiovascular benefits of these drugs remain limited and have been a subject of ongoing research.

Methotrexate is among the most frequently utilized conventional synthetic DMARDs in the treatment of PsA [143]. While there is evidence to suggest that methotrexate can reduce systemic inflammation and improve joint symptoms, its role in cardiovascular health remains a subject of debate. Some studies have suggested that methotrexate can have a beneficial effect on cardiovascular outcomes by reducing markers of inflammation, like CRP and TNF-α, while others have raised concerns about its potential to increase the risk of cardiovascular events in certain patients, particularly with long-term use [144,145].

Sulfasalazine and leflunomide, other second-line conventional synthetic DMARDs, have been demonstrated to reduce disease activity and systemic inflammation [146]. However, the impact of these medications on cardiovascular health is indirect, and further research is necessary to fully elucidate their effects in this regard.

#### 7.3.4. Biologic Disease-Modifying Antirheumatic Drugs (DMARDs)

Biologic DMARDs have emerged as a substantial advancement in the management of PsA, demonstrating potential for mitigating cardiovascular risk by targeting specific cytokines implicated in both inflammation and atherosclerosis [147]. These therapeutic agents have been demonstrated to markedly reduce systemic inflammation, a pivotal element in the pathogenesis of cardiovascular complications.

TNF-α inhibitors, including etanercept, adalimumab, and infliximab, are among the most utilized biologic DMARDs in the treatment of PsA [148]. TNF-α, a pro-inflammatory cytokine, plays a central role in joint inflammation and is implicated in the pathogenesis of atherosclerosis [149]. Some researchers have demonstrated that TNF-α inhibitors can effectively reduce both inflammation and the burden of atherosclerotic plaque, thereby potentially enhancing vascular health [150]. Moreover, TNF-α inhibitors have been associated with improved endothelial function, a critical factor in reducing cardiovascular risk [151].

Secukinumab and ixekizumab are biologics that target IL-17, another cytokine involved in PsA and vascular inflammation [152]. While their primary indication is to reduce joint inflammation and improve skin manifestations, these therapies may have beneficial effects on vascular inflammation and atherosclerotic progression [153]. The evidence supporting the cardiovascular benefits of IL-17 inhibitors is still limited, but early studies suggest potential improvement in vascular health and plaque stability [153]. Bimekizumab, a novel IL-17A/F inhibitor, has shown promising efficacy in PsA by targeting both IL-17A and IL-17F, which may provide enhanced suppression of inflammation. Its potential impact on cardiovascular outcomes is an area of ongoing research [154].

Guselkumab and tildrakizumab, which are biologic agents that inhibit IL-23, another implicated cytokine in the pathogenesis of PsA and atherosclerosis, are also being studied [155]. The cardiovascular effects of these inhibitors are not fully understood; however, emerging research suggests that they may reduce vascular inflammation and atherosclerotic plaque formation.

#### 7.3.5. Janus Kinase (JAK) Inhibitors

JAK inhibitors, including tofacitinib and baricitinib, are oral medications that target intracellular signaling pathways that play a role in the immune response [155]. JAK inhibitors have demonstrated notable efficacy in the management of joint inflammation in patients with PsA [156]. JAK inhibitors are recommended in patients refractory to NSAIDs, conventional DMARDs, and biologic DMARDs. While their cardiovascular benefits are less well-established, JAK inhibitors may improve vascular function by reducing systemic inflammation, a key contributor to cardiovascular disease. However, concerns regarding their safety profile, particularly their potential to increase the risk of venous thromboembolism (VTE) and major adverse cardiovascular events (MACE), require careful monitoring [136,157].

## 8. Future Directions

While contemporary therapeutic strategies have proven efficacious in the management of joint inflammation and cardiovascular risk, the emergence of novel treatments and future research directions hold promise for further enhancing patient outcomes. Precision medicine is the utilization of individualized treatment regimens based on patient’s genetic profile, disease subtype, and particular cardiovascular risk factors [158]. Moreover, the combination of TNF-α inhibitors or IL-17 inhibitors with statins or antihypertensive medications could yield synergistic benefits in reducing both systemic inflammation and cardiovascular risk [159]. Therefore, the implementation of precision medicine will have a role to play in the selection of appropriate treatment for PsA in the future.

The advent of novel therapeutic agents that target metabolic dysfunction, exemplified by GLP1-RAs and SGLT2i, has garnered significant attention due to their cardiovascular benefits beyond glucose lowering effects [160]. These therapeutic modalities hold promise for use in patients with PsA who also have obesity or T2DM.

Gene therapy and advanced immunomodulation techniques have the potential to offer novel therapeutic approaches to the treatment of PsA and the reduction of cardiovascular risk [161]. By targeting specific genes or pathways associated with both inflammation and atherosclerosis, these treatments have the potential to offer more precise and effective solutions. Though still investigational, gene therapy and immunomodulation show great promise for advancing PsA and cardiovascular disease management.

## 9. Conclusions

In summary, PsA represents a multifaceted disease that extends far beyond joint and skin manifestations, posing a significant yet underrecognized cardiometabolic risk. Chronic systemic inflammation, mediated by cytokines, such as TNF-α, IL-6, and IL-17, plays a pivotal role in metabolic dysregulation, exacerbating insulin resistance, adipokine imbalance, and lipid dysfunction. These factors collectively heighten the risk of CVD, contributing to increased morbidity and mortality in PsA patients. The intricate interplay between inflammation, oxidative stress, and metabolic abnormalities underscores the need for a paradigm shift in PsA management—one that integrates rheumatologic, metabolic, and cardiovascular considerations.

Early screening, risk stratification, and a multidisciplinary approach to treatment are essential for mitigating long-term complications. By incorporating targeted anti-inflammatory therapies with lifestyle and pharmacologic interventions addressing metabolic dysfunction, clinicians can optimize patient outcomes. Future research should prioritize identifying novel biomarkers and therapeutic targets that bridge immune and metabolic pathways, ultimately refining precision medicine approaches in PsA. Recognizing and addressing the cardiometabolic burden of PsA is paramount to improving both disease prognosis and overall patient health.

## Figures and Tables

**Table 1:** Summary of adipokine characteristics in psoriatic arthritis.

Adipokine	Role in PsA	Cardiovascular Risk Impact	Associated Effects
Leptin	Pro-inflammatory, regulates energy balance	Elevated levels correlate with increased cardiovascular risk, endothelial dysfunction, and vascular pathology in PsA.	Induces cytokine production (e.g., TNF-α, IL-6), endothelial dysfunction, angiogenesis, disease activity correlation.
Adiponectin	Anti-inflammatory, cardioprotective	Low levels linked to increased disease activity, subclinical myocardial dysfunction, and early cardiometabolic risk.	Enhances insulin sensitivity, inhibits smooth muscle cell proliferation, reduces endothelial adhesion.
Resistin	Pro-inflammatory, linked to insulin resistance	Elevated levels associated with metabolic syndrome components (e.g., hyperglycemia, dyslipidemia) and low-grade inflammation.	Correlates with disease severity in PsA, though direct link to subclinical atherosclerosis unclear.

Abbreviations: PsA—psoriatic arthritis, TNF-α—tumor necrosis factor alpha, IL-6—interleukin 6.

## Data Availability

No new data were created or analyzed in this study.
